# Systematic review and meta-analysis: cholecystectomy and the risk of cholangiocarcinoma

**DOI:** 10.18632/oncotarget.19570

**Published:** 2017-07-26

**Authors:** Jianping Xiong, Yaqin Wang, Hanchun Huang, Jin Bian, Anqiang Wang, Junyu Long, Ying Zheng, Xinting Sang, Yiyao Xu, Xin Lu, Haitao Zhao

**Affiliations:** ^1^ Department of Liver Surgery, Peking Union Medical College Hospital, Chinese Academy of Medical Sciences and Peking Union Medical College (CAMS & PUMC), Beijing, China; ^2^ Department of Interventional Radiology, The First Affiliated Hospital of China Medical University, Shenyang, China; ^3^ State Key Laboratory of Quality Research in Chinese Medicine, Institute of Chinese Medical Science, University of Macau, Macau SAR, China

**Keywords:** cholecystectomy, cholecystolithiasis, cholangiocarcinoma, biliary tract neoplasms, meta-analysis

## Abstract

Studies have reported that cholecystectomy may increase the risk of cholangiocarcinoma. However, this association is controversial. Thus, we conducted a systematic review and meta-analysis to explore the relationship between cholecystectomy and the risk of cholangiocarcinoma. Relevant studies were identified by searching PubMed, EMBASE, ISI Web of Science published before February 2017. We used the random effects model proposed by DerSimonian and Laird to quantify the relationship between cholecystectomy and risk of cholangiocarcinoma. Publication bias was evaluated using funnel plots, Begg's and Egger's tests. Subgroup and sensitivity analyses were performed to validate the stability of the results. 16 articles, comprising 220,376 patients with cholecystectomy and 562,392 healthy controls, were included in our research. Our meta-analysis suggested that the risk of cholangiocarcinoma was significantly higher in the cholecystectomized patients in comparison with healthy controls, with heterogeneity among studies (summary odds ratio [OR] = 0.72; confidence interval [CI] = 0.55–0.90; I^2^ = 69.5%). Additionally, this association was also observed in cohort studies (OR = 0.83; 95% CI = 0.73–0.94) and case-control studies (OR = 0.60; 95% CI = 0.40–0.80). However, When the intrahepatic cholangiocarcinoma and extrahepatic cholangiocarcinoma were analyzed separately, the present study only indicated cholecystectomy was associated with increased the risk of extrahepatic cholangiocarcinoma (OR = 1.19; 95% CI = 0.32–2.05), rather than intrahepatic cholangiocarcinoma (OR = 1.19; 95% CI = 0.32–2.05). In conclusion, cholecystectomy was associated with a significant 54% increase in the risk of cholangiocarcinoma, especially in the extrahepatic cholangiocarcinoma.

## INTRODUCTION

Gallstones are abnormal masses of a solid mixture of cholesterol crystals, mucin, calcium bilirubinate, and proteins [1]. Gallstone is the most common gastrointestinal disease. An estimated 10% of Europeans and Americans are carriers of gallbladder stones [2]. Furthermore, along with the improvement of living standards and population overall life extension, the incidence of cholecystolithiasis seems to be increasing [3, 4]. Additionally, gallstone is the most expensive gastrointestinal diseases, and become a global health burden [5]. For example, it costs of $6.5 billion approximately annually in the U.S [6]. Most of gallstones are silent. However, around 25% of gallstones are symptomatic and accompanied with severe complications, which need to remove the gallbladder by surgically, usually by laparoscopic cholecystectomy [7, 8]. An estimated 700,000 cholecystectomies are conducted annually in the US [3]. Over the past few decades, cholecystectomy has been reported to increase the risk of some types of cancer, including colorectal cancer, liver cancer and pancreatic cancer [9–14]. Recently, studies reported cholecystectomy may increase the risk of cholangiocarcinoma. However, this association is controversial [15–20].

Cholangiocarcinoma, which was first described by Durand-Fardel in 1840, is a malignant tumor originating from bile duct epithelium [21]. Cholangiocarcinoma is the second commonest primary liver cancer, as it accounts for 10%–25% of liver malignant tumors and 3% of all gastrointestinal neoplasms [22, 23]. Moreover, the incidence of cholangiocarcinoma still has been increasing over the past few decades. Somewhat surprisingly, the epidemiological characteristics between intrahepatic cholangiocarcinoma (ICC) and extrahepatic cholangiocarcinoma (ECC) are different, the incidence of ICC have been increasing; On the contrary, the incidence of ECC have been declining in some parts of the world, such as UK and USA [24]. In the United States, the age-adjusted incidence of ICC increased by 165%, whereas ECC declined by 14% during the past two decades [25]. Besides, the prognosis of cholangiocarcinoma is particularly poor. The overall 1-, 3- and 5-year relative survival rates are reportedly 25.0%, 9.7% and 6.8%, and almost no changes in recent decades [26]. Thus, to better understand the relationship between cholecystectomy and the risk of cholangiocarcinoma, we conducted a systematic review with meta-analysis of published observational studies.

## RESULTS

### Study selection and study characteristics

**Figure 1:**
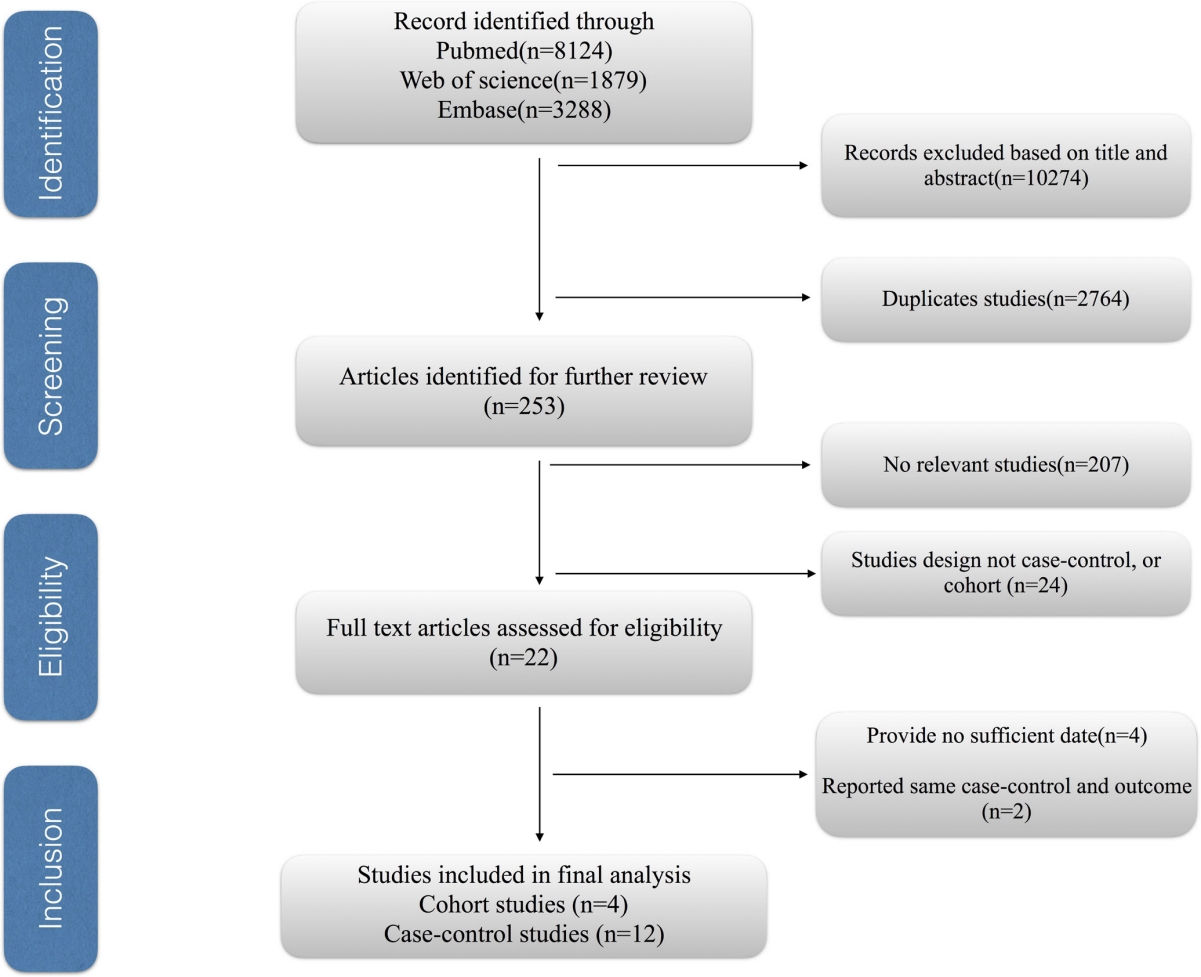
The process of study selection for the meta-analysis

Figure 1 shows the process of selecting studies. We obtained 13291 articles through the initial search (8124 from PubMed, 1879 from EMBASE, 3288 from Web of Science), 3120 of which were duplicates. We excluded a further 10274 studies based on title and abstract review. Finally, four studies were further excluded due to providing insufficient information [27–30], we identified 16 eligible observational articles for our meta-analysis [15–20, 31–40].

The main characteristics of the included studies are listed in Table 1. Six studies were performed in China, four in the USA, two in Denmark, one in Greece, one in Swedish, one in Korea and one in Taiwan. All included studies were observational studies and included 12 case-control studies and four cohort studies. The meta-analysis included 220,376 patients with cholecystectomy and 562,392 healthy controls to investigate the effect of cholecystectomy on the risk of cholangiocarcinoma. The data collected in the study ranged from 1965 to 2014. The NOS scores of the included studies ranged from 5 to 9, with 12 high quality studies and only four of medium quality (Supplementary Tables 1 and 2).

**Table 1:** The main characteristics of the included studies

Study/Years of Publication	Country	No. Case/control	Follow	Sources of Controls	Subtype of cancer	Subtype of study	Adjusted Factors	Adjusted OR/RR (95% CI)
Lee.2015	korea	276/452	2007–2013	Hospital	CC	Case-control	Cigarette smoking, heavy alcohol consumption, obesity, choledocholithiasis, cholecystolithiasis, hepatolithiasis, ulcerative colitis, alcoholic liver disease, thyroid disease, chronic pancreatitis, pypertension, diabetes mellitus, HBV infection, HCV infection, liver fluke infestation	1.38(0.67, 2.84)
Zhang.2014	China	127/254	1993–2013	Hospital	ICC	Case-control	Age, sex, BMI, Smoking, Alcohol consumption, HBV infection, HCV, Liver cirrhosis.	1.53(0.52, 4.49)
Chow.1999	Denmark	17715/42461	1977–1989	Population	CC	Cohort	age and gender	1.12(0.81, 1.43)
WELZEL.2007	USA	1084/102782	1993–1999	Population	CC	Case-control	age, sex, race/ethnicity, cholecochal cysts, cholangitis, biliary cirrhosis, cholelithiasis, cholecystolithiasis, choledocholithiasis, liver flukes, alcoholic liver disease, nonspecific cirrhosis, HCV infection, diabetes mellitus type II, crohn’s disease, ulcerative colitis, duodenal ulcer, chronic pancreatitis, smoking, obesity	5.4(3.9, 7.5)
Chen.2014	Taiwan	5850/62180	2000–2014	Population	ECC	Cohort	sex, age and number of comorbidities	2.22(0.91, 5.41)
Tao.2009	China	188/380	1998–2008	Hospital	CC	Case-control	age, gender, diabetes mellitus	3.6(0.9, 15.1)
WELZEL.2006	Denmark	764/3056	1978–1991	Population	ICC	Case-control	Alcoholic liver diseases, nonspecific cirrhosis, cholangitis, choledocholithiasis, inflammatory bowel disease, diabetes, obesity	1.56(0.65, 3.73)
Cai.2011	China	313/608	2000–2004	Hospital	ECC1	Case-control	choledocholithiasis, hepatolithiasis, cholecystolithiasis, biliary ascariasis, liver fluke and liver schistosomiasis were the risk factors for HC, while HBV infection, HCV infection, PSC, UC, alcoholic liver disease, type II diabetes mellitus, alcohol and smoking	7.01(1.90, 25.95)
Zhou.2013	China	239/478	1999–2011	Hospital	ECC	Case-control	sex, age (as continuous variable), liver cirrhosis, cholecystolithiasis, choledocholithiasis, hepatolithiasis, diabetes mellitus and family history of other cancer.	4.04(1.58, 10.31)
CHALASANI.2000	USA	26/87	1991–1998	Hospital	CC	Case-control	PSC and geographic location.	7.11(2.71, 18.67)
Liu.2011	China	87/288	2000–2008	Hospital	CC	Case-control	HBV infection, HCV infection, and liver fluke infestation, Diabetes mellitus, hypertension, alcohol, smoking,	0.76(0.46, 1.24)
Kuper.2001	Greece	6/360	1995–1998	Hospital	CC	Case-control	Years of schooling, tobacco smoking, excessive alcohol consumption or coffee drinking	2.39(0.27, 21.22)
Shaib.2007	USA	248/236	1992–2002	Hospital	CC	Case-control	race, age, gender, HCV, HBV markers, and mild/moderate alcohol drinking.	1.1 (0.6, 2.2)
Nogueira.2014	USA	118/3681	1992–2005	Population	CC	Cohort	age and gender	1.19(0.98, 1.43)
Nordenstedt.2012	Swedish	192960/345251	1965–2008	Population	CC	Cohort	age, sex and gender	1.28(1.14, 1.43)
Peng.2011	China	98/126	2002–2009	Hospital	ICC	Case-control	HBV infection, cirrhosis, hepatolithiasis, choledocholithiasis, cholecystolithiasis, and liver fluke infestation, Diabetes mellitus, Hypertension	1.08 (0.42, 2.81)

ICC, intrahepatic cholangiocarcinoma. ECC, extrahepatic cholangiocarcinoma. HBV, hepatitis B virus. HCV, hepatitis C virus. RR, relative risk. OR, odds ratio. CI, confidence interval.

**Table 2:** Subgroup and sensitivity analyses of the effect of cholecystectomy and the risk of cholangiocarcinoma

Subgroup	No. of studies	RR (95%CI)	I2 value(%)	*P* value
**All studies**	16	1.54 (1.15, 1.94)	86.3	0.001
**Subtype of cancer**				
ECC	9	2.31 (1.34, 3.28)	86.3	0.001
ICC	10	1.40 (0.94, 1.87)	68.2	0.001
**Geographic areas**				
West	8	1.71 (1.19, 2.23)	88.8	0.001
East	8	1.17 (0.65, 1.69)	16.7	0.298
**Study deign**				
Cohort study	4	1.24 (1.12, 1.35)	0	0.618
Case-control study	12	2.31 (1.23, 3.39)	84.7	0.001
**Adjustment for confoundersLiver fluke infestation**				
Yes	5	2.68 (0.53, 4.82)	94.0	0.001
No	11	1.24 (1.13, 1.35)	0	0.695
**Cholangitis**				
Yes	4	5.12 (0.64, 9.59)	88.9	0.001
No	12	1.21 (1.10, 1.31)	0	0.449
**Gallstone**				
Yes	6	3.09 (0.80, 5.39)	89.7	0.001
No	10	1.17 (1.02, 1.32)	17.6	0.281
**Smoking**				
Yes	6	2.83 (0.51, 5.16)	92.5	0.001
No	10	1.24 (1.13, 1.35)	0	0.610
**Alcohol intake**				
Yes	8	2.24 (0.96, 3.53)	89.5	0.001
No	8	1.24 (1.13, 1.35)	0	0.431
**Sensitive analyses**				
High quality studies	12	1.73 (1.19, 2.28)	84.6	0.001
**Fixed-effects vs random-effects model method**				
Fixed-effects model	16	1.24 (1.13, 1.34)	76.3	0.001
Random-effects model	16	1.54 (1.15, 1.94)	79.7	0.001

ICC, intrahepatic cholangiocarcinoma. ECC, extrahepatic cholangiocarcinoma. RR, relative risk; CI, confidence interval

### Association between cholecystectomy and the risk of cholangiocarcinoma

**Figure 2:**
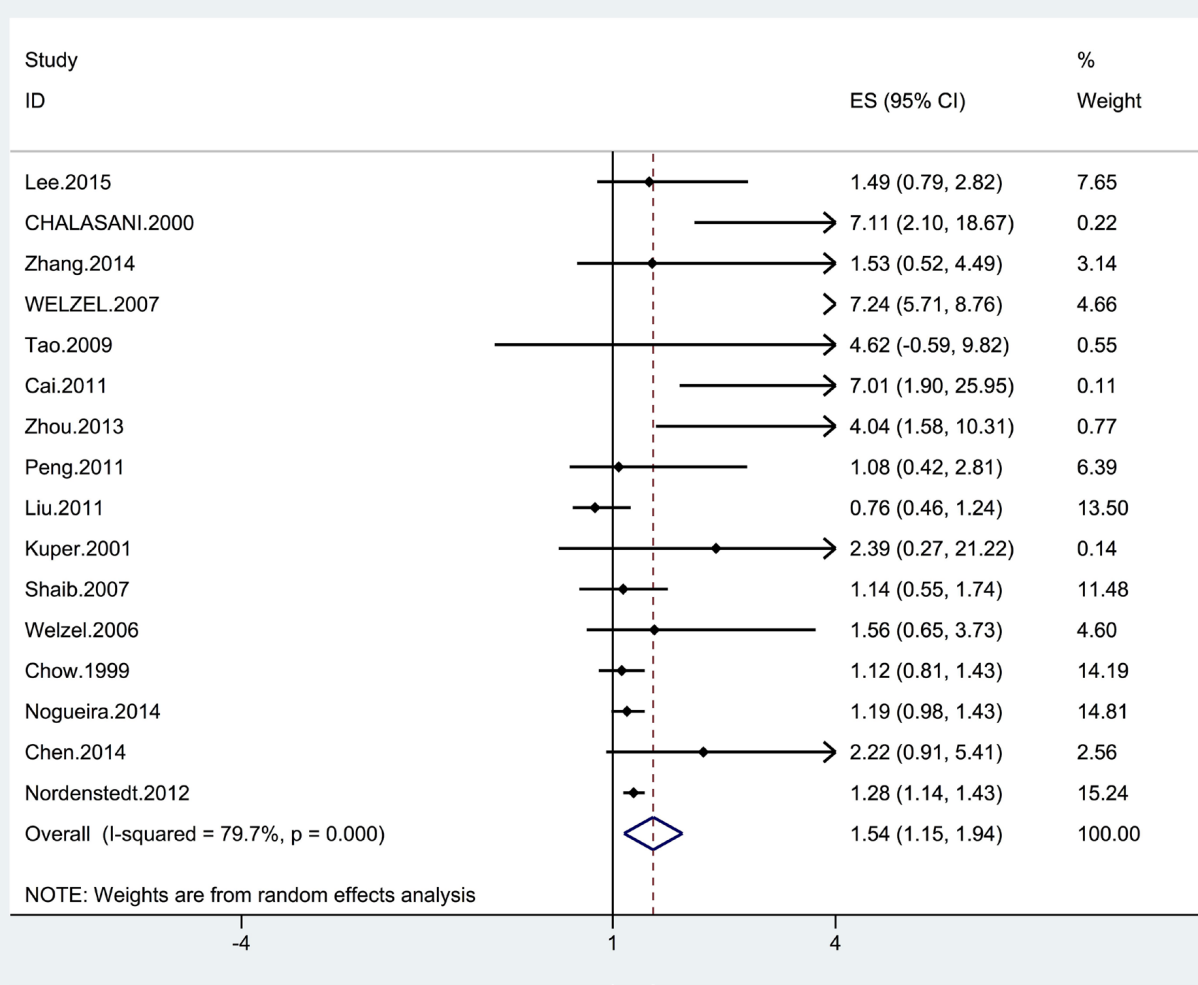
Forrest plot showing the relationship between cholecystectomy and the risk of cholangiocarcinoma Points represent the risk estimates for each individual study. Horizontal lines represent 95% confidence intervals, and diamonds represent the summary risk estimates with 95% confidence intervals. ICC, intrahepatic cholangiocarcinoma. ECC, extrahepatic cholangiocarcinoma. CI, confidence interval. ES, effect size.

Four cohort and 12 case-control studies were included to investigate the relationship between cholecystectomy and the risk of cholangiocarcinoma. Six studies reported significantly higher risk of cholangiocarcinoma in patients who had cholecystectomies in comparison with the healthy controls. Only one studies reported cholecystectomy was associated with a decreased risk of cholangiocarcinoma. The remaining of the studies did not show a relationship. The pooled estimate was significant (OR = 1.54; 95% CI = 1.15–1.94), with significant heterogeneity (I^2^ = 69.5%; *p* = 0.006) (Figure 2). The present study indicated a 54% increase in the risk for cholangiocarcinoma among the cholecystectomized patients in comparison with healthy controls. However, this relationship was only observed in ECC (OR = 2.31; 95% CI = 1.34–3.28, I^2^ = 86.3%), rather than ICC (OR = 1.40; 95% CI = 0.94–1.87, I^2^= 68.2%) (Table 2).

### Subgroup and sensitivity analyses

**Figure 3:**
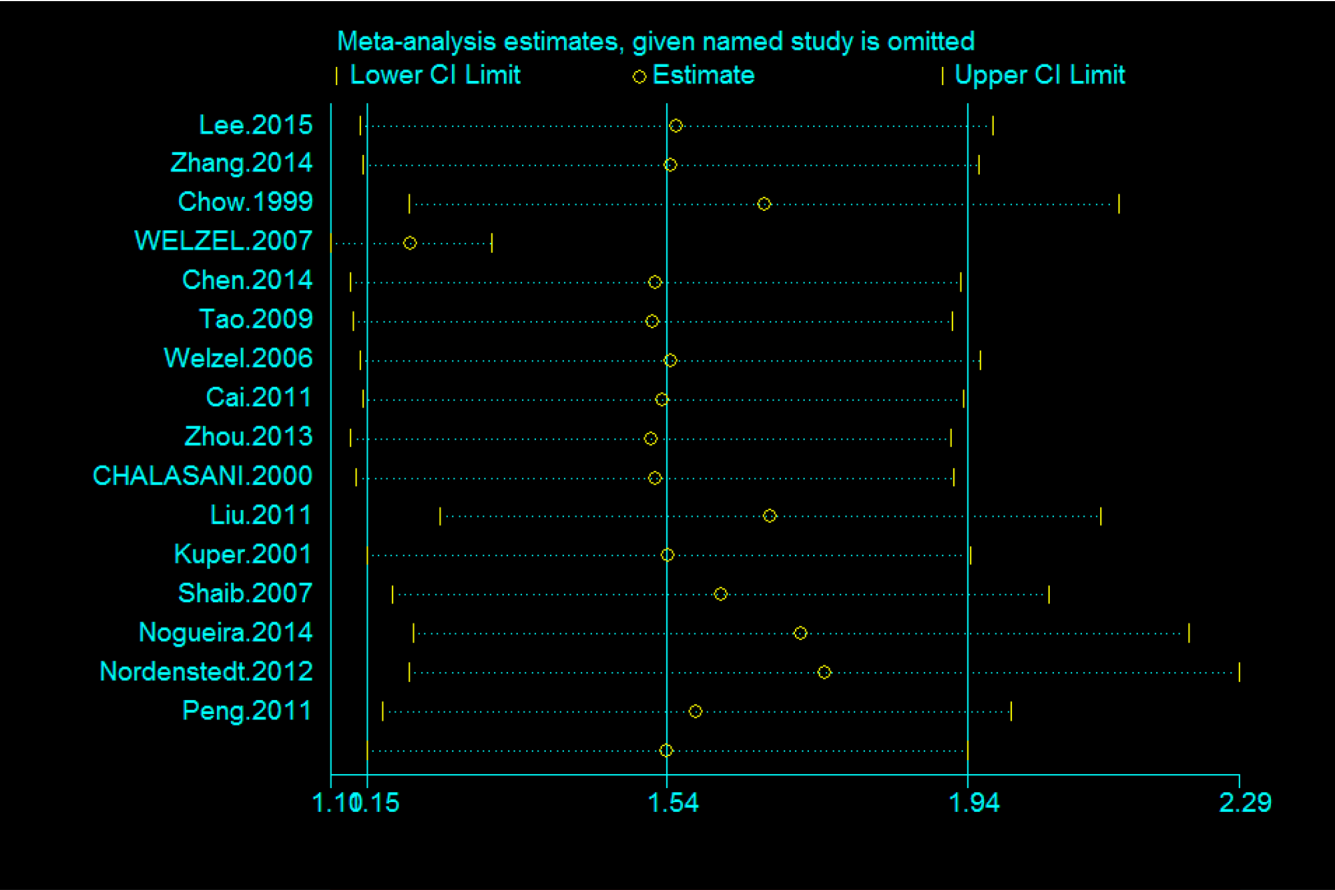
Sensitivity analysis of the association between cholecystectomy and the risk of cholangiocarcinoma

The results of the subgroup analyses and sensitivity analyses are shown in Table 2. When the studies from Western countries (USA, Denmark, Greece and Swedish) and Eastern countries (Taiwan, Korea and China) were analyzed, a significant difference was found between the two areas. Patients with cholecystectomy in western countries were more likely to develop cholangiocarcinoma compared to eastern countries (western countries: OR = 1.71; 95% CI = 1.19–2.23 and eastern countries: OR = 1.17; 95% CI = 0.65–1.69) (Table 2). According to the sensitivity analyses, despite excluding studies that the NOS sources were < 7, the relationship between cholecystectomy and the risk of cholangiocarcinoma remained stable (Table 2). Additionally, the overall results for the relationships of cholecystectomy to cholangiocarcinoma were maintained when the pooling model was altered (fixed-effects model: OR = 1.24; 95% CI = 1.13–1.34 and random-effects model: OR = 1.54; 95% CI = 1.15–1.94) (Table 2). Besides, when we sequentially excluded one study in one turn to assess the stability of the results, no study could possibly affect the pooled risk estimate (Figure 3).

### Publication bias

**Figure 4:**
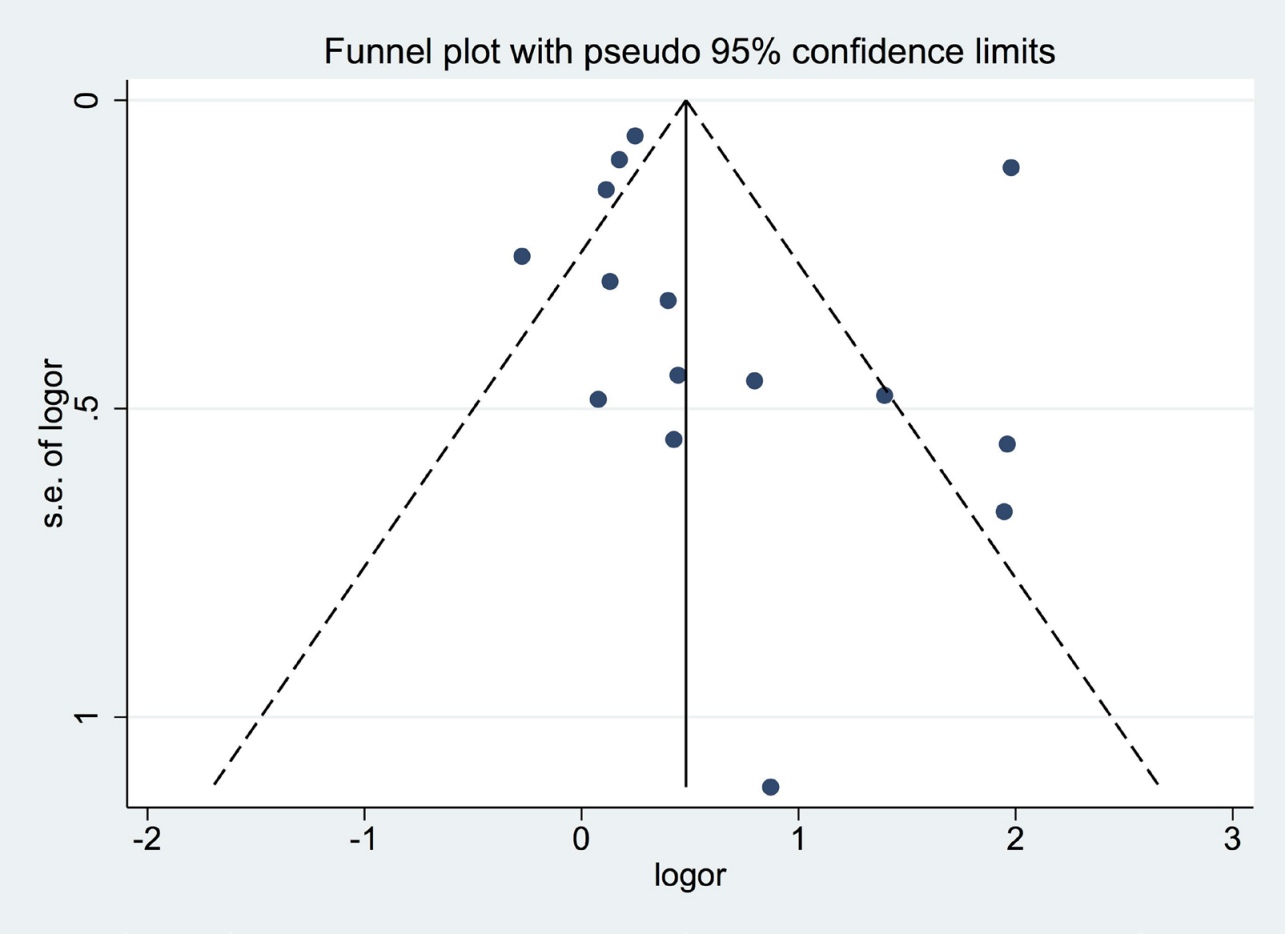
Funnel plot of studies included in the meta-analysis of the relationships between cholecystectomy and the risk of cholangiocarcinoma Logor: Log odds ratio. SE: standard error.

The funnel plot did not reveal substantial asymmetry. Additionally, Begg's and Egger's tests did not identify substantial publication bias (*p* > 0.05) (Figure 4).

## DISCUSSION

The causes of cholangiocarcinoma remain poorly understood. Only a few risk factors for the disease have been identified. These include primary sclerosing cholangitis, hepatolithiasis, bile-duct cysts and parasitic infections [41]. Many recent meta-analyses have identified additional factors that may affect the risk of cholangiocarcinoma, including hepatitis B or C, obesity, diabetes mellitus, cirrhosis, alcohol consumption, smoking [42–46]. To our knowledge, this is the first comprehensive meta-analysis to investigate the relationship between cholecystectomy and the risk of cholangiocarcinoma. 16 studies were identified to examine the effect of cholecystectomy on the risk of cholangiocarcinoma and found that the risk of cholangiocarcinoma was significantly higher in 220,376 patients with cholecystectomy compared with 562,392 healthy populations (OR = 0.72; 95% CI = 0.55–0.90), with significant heterogeneity among studies. This effect also was observed in cohort and case-control studies. When the analysis was stratified by geographic area, this effect was more pronounced in Eastern countries in comparison with Western countries. However, When the ICC and ECC were analyzed separately, the present study only indicated cholecystectomy was associated with increased risk of ECC (OR = 1.19; 95% CI = 0.32–2.05), rather than ICC (OR = 1.19; 95% CI = 0.32–2.05).

Our study only demonstrated an association between cholecystectomy and an increased risk of cholangiocarcinoma; the data cannot establish a causative role for cholecystectomy in this regard. However, if such a causative role is present, possible mechanisms could be the following. First, several authors consider it as the effect of gallstones, rather than the ensuing cholecystectomy, which results in cancer. Early study indicated gallstones may increase the risk of cholangiocarcinoma, especially in ECC [15, 47, 48]. Second, removal of the gallbladder leads to the accumulation of bile and secondary bile acids, and secondary bile acids was associated with increased in the presence of gallstones [49–51]. Additionally, previous studies also reported secondary bile acids can enhance tumor formation in the liver [52]. Because both cholangiocytes and hepatocytes differentiate from the same progenitor cells, similar to the carcinogenesis of hepatocytes [53], secondary bile acids might induce carcinogenesis in cholangiocytes through the same mechanism.

Our study has several strengths. First, it is the first meta-analysis with a large sample size (220,376 patients with cholecystectomy and 562,392 healthy populations) to evaluate the effect of cholecystectomy on the risk of cholangiocarcinoma. Therefore, the findings may provide us insight into the relationship between cholecystectomy and the risk of cholangiocarcinoma, and these results are of potential interest to the field of cholangiocarcinoma research. Secondly, subgroup and sensitivity analyses were performed to determine the factors that may affect results. It makes our findings more reliable. Third, we performed a comprehensive literature search of the PubMed, EMBASE and Web of Science databases to identify potential studies to investigate the relationships between cholecystectomy and the risk of cholangiocarcinoma. In addition, most of the studies included in our meta-analysis were of high quality. All of these characteristics make the conclusions of our study more convincing.

There are several limitations that should be considered. First, most of the studies included in our meta-analysis were case-control studies, which was prone to generate recall and selection biases. Additionally, the heterogeneity among studies was significant because of different study designs and demographic characteristics inconsistency. Second, the present study only investigated the risk of cholangiocarcinoma in patients with cholecystectomy compared with healthy population. As a result of the restricted number of included studies in the analysis, the risk of cholangiocarcinoma in patients with cholecystectomy compared with gallstone patients was not explored. Third, what is being observed is just an association, which is subject to confounding bias. The established risk factors for cholangiocarcinoma include primary sclerosing cholangitis, hepatolithiasis, bile-duct cysts and parasitic infections [41]. However, only a few studies adjusted it in their models. Besides, the results of the present study are subject to diagnostic bias. Patients had cholecystectomies are more likely to undergo physical examination and thus might be more likely to have cholangiocarcinoma detected early. Finally, the length of time necessary following a cholecystectomy for any carcinogenic effect to have occurred remains unknown. It is likely that some cases of cholangiocarcinoma included in this research occurred too soon after cholecystectomy [39, 40].

In summary, our meta-analysis indicated that the risk of cholangiocarcinoma was associated with a 54% increase in patients who had cholecystectomies in comparison with healthy controls, and the relationship was also demonstrated in cohort and case-control studies. However, When the ICC and ECC were analyzed separately, the present study only indicated cholecystectomy was associated with increased the risk of ECC, rather than ICC. More prospective studies and basic research are still needed to validate the association of cholecystectomy and cholangiocarcinoma risk and the potential mechanisms.

## MATERIALS AND METHODS

### Data sources and search strategy

We searched published reports in the PubMed, EMBASE and Web of Science databases using the following keywords: (“gallstone” OR “cholelithiasis” OR “cholecystolithiasis” OR “choledocholithiasis” OR “cholecystectomy” OR “gallbladder surgery”) and (“biliary tract cancer” OR “bile duct cancer” OR “biliary tract neoplasms” OR “cholangiocarcinoma”). We placed no restrictions on the language or date of publication.

### Eligibility criteria for study selection

The eligibility criteria were as follows: study design (case control or cohort); cholecystectomy as the exposure factor and cholangiocarcinoma or bile duct cancer or biliary tract cancer as the outcome; and odds ratio (OR)/risk ratio (RR) values and corresponding 95% confidence intervals available or sufficient information to calculate them. If two studies reported the same data, we selected the study with the larger sample.

### Data abstraction and quality assessment

Two researchers (Y.W. and A.W.) independently extracted the required information from the selected studies in a standardized manner. We collected the following information from each article: first author's name, year of publication, country of origin, study design (case-control or cohort), number of participants, duration of follow-up, sources of controls, adjustment for confounding variables, and OR/RR values and 95% CIs.

The Newcastle-Ottawa Scale (NOS) [54] was used to evaluate the quality of the included studies. We assigned quality categories according to the scores of each study. Specifically, NOS scores of <4, 4–6, and 7–9 indicated low-, medium-, and high-quality studies, respectively [55]. The maximum total score was 9 points. We resolved discrepancies by consensus.

### Statistical analyses

The OR/RR values and corresponding 95% CIs were used to evaluate the risk of cholangiocarcinoma in with a history of cholecystectomy. We treated hazard ratios as equivalent to RRs. We used the random effects model proposed by DerSimonian and Laird to quantify the relationship between cholecystectomy and the risk of cholangiocarcinoma [56].

The I^2^ statistic was used to quantify the heterogeneity between studies, and I^2^ values of 25%, 50%, and 75% represented low, medium, and high heterogeneity, respectively [57]. *P* values less than 0.1 indicated that clear heterogeneity existed. Publication bias was qualified with funnel plot and Begg's [58] and Egger's [59] tests, and funnel plot asymmetry and *P* values less than 0.05 indicated the presence of bias.

We also performed subgroup analyses by subtype of cancer, geographic areas, study design and whether liver fluke infestation, cholangitis, gallstones, alcohol intake or smoking were adjusted for in the models. Sensitivity analysis was conducted to assess the stability of the results by sequentially excluding one study in one turn. Additionally, sensitivity analyses were also performed by changing the pooling model (random-effects model or fixed-effects model) and excluded studies that the NOS sources were < 7.

All statistical analyses were performed using STATA version 12.0 (Stata).

## SUPPLEMENTARY MATERIALS TABLES



## Abbreviations

ICC: intrahepatic cholangiocarcinoma
ECC: extrahepatic cholangiocarcinoma
