# Supplementary material for: Systematic review and meta-analysis: cholecystectomy and the risk of cholangiocarcinoma
Source: Oncotarget. 2017 Jul 26;8(35):59648–57. doi: 10.18632/oncotarget.19570 (PMC5601764; doi:10.18632/oncotarget.19570)
Supplement: Supplementary file 1 [file oncotarget-08-59648-s001.pdf]

Systematic review and meta-analysis: cholecystectomy and the risk of cholangiocarcinoma

SUPPLEMENTARY MATERIALS

Supplementary Table 1: Scores of the newcastle-ottawa scale for cohort studies

Study/Years of Publication	representativeness of exposed cohort	Selection of the non-exposed cohort	Determination of exposure	outcome not present at study start	Controlling the important factors or confounding factors	Assessment of outcome	Follow-up long enough for outcome to occur	Integrity of follow up	Total score
Nogueira.2014	*	*	*		*	*		*	6
Nordenstedt.2012	*	*	*		**	*	*	*	8
Chow.1999	*	*		*	**	*		*	7
Nechuta .2012	*	*	*	*	*	*	*	*	8

Supplementary Table 2: Scores of the newcastle-ottawa scale for case control studies

Study/Years of Publication	Fully defined cases	Representative cases	Selection of controls	Definition of controls	Controlling the important factors or confounding factors.	Determination of exposure	Same method of determination for cases and control	Non-response rate	Total score
Lee.2015	*	*		*	**		*		6
Zhang.2014	*	*	*		**	*	*		7
WELZEL.2007	*	*		*	**	*	*	*	8
Tao.2009	*	*		*	**	*	*	*	8
WELZEL.2006	*	*	*	*	**	*	*		8
Cai.2011	*	*	*		**		*	*	7
Zhou.2013	*	*	*		**		*	*	7
CHALASANI.2000	*			*	*	*		*	5
Liu.2011	*	*		*	**	*	*	*	8
Kuper.2001	*	*	*		**		*		6
Shaib.2007	*	*	*		**		*	*	7
Peng.2011	*	*		*	**	*	*	*	8
